# Dairy Cow Mastitis Detection by Thermal Infrared Images Based on CLE-UNet

**DOI:** 10.3390/ani13132211

**Published:** 2023-07-05

**Authors:** Qian Zhang, Ying Yang, Gang Liu, Yuanlin Ning, Jianquan Li

**Affiliations:** 1 College of Information and Electrical Engineering, China Agricultural University, Beijing 100083, China; s20203081477@cau.edu.cn (Q.Z.); pac@cau.edu.cn (G.L.); lijianquan@cau.edu.cn (J.L.); 2 Key Laboratory of Modern Precision Agriculture System Integration Research, Ministry of Education, Beijing 100083, China; 3 Key Laboratory of Agricultural Information Acquisition Technology, Ministry of Agriculture, College of Information and Electrical Engineering, China Agricultural University, Beijing 100083, China

**Keywords:** thermal image, cow mastitis detection, ellipse fitting, UNet, livestock precision farming

## Abstract

**Simple Summary:**

Mastitis is one of the most serious diseases in dairy husbandry, and its timely detection is critical for improving the efficiency of treatment and reducing breeding risks. However, the traditional “contact-based” manual detection method is complex and unsuitable for large-scale production practices. In recent years, the rapid development of deep learning technology has brought new possibilities. We present a novel approach for cow mastitis detection based on thermal infrared image segmentation technology. By automatically segmenting the key parts of the cow’s eyes and udders in the thermal infrared image, it becomes possible to determine mastitis based on temperature. The results show that this method can meet the requirements of the timely and accurate detection of cow mastitis in large-scale dairy farms.

**Abstract:**

Thermal infrared technology is utilized for detecting mastitis in cows owing to its non-invasive and efficient characteristics. However, the presence of surrounding regions and obstacles can impede accurate temperature measurement, thereby compromising the effectiveness of dairy mastitis detection. To address these problems, we proposed the CLE-UNet (Centroid Loss Ellipticization UNet) semantic segmentation algorithm. The algorithm consists of three main parts. Firstly, we introduced the efficient channel attention (ECA) mechanism in the feature extraction layer of UNet to improve the segmentation accuracy by focusing on more useful channel features. Secondly, we proposed a new centroid loss function to facilitate the network’s output to be closer to the position of the real label during the training process. Finally, we used a cow’s eye ellipse fitting operation based on the similarity between the shape of the cow’s eye and the ellipse. The results indicated that the CLE-UNet model obtained a mean intersection over union (MIoU) of 89.32% and an average segmentation speed of 0.049 s per frame. Compared to somatic cell count (SCC), this method achieved an accuracy, sensitivity, and F1 value of 86.67%, 82.35%, and 87.5%, respectively, for detecting mastitis in dairy cows. In conclusion, the innovative use of the CLE-UNet algorithm has significantly improved the segmentation accuracy and has proven to be an effective tool for accurately detecting cow mastitis.

## 1. Introduction

According to the United States Department of Agriculture (USDA), the global dairy cattle population increased by 1.1% year-on-year in 2021, reaching 140,642,000 cows in 2022 [1]. This growth creates a vast development space for the global dairy farming industry. However, mastitis, one of the most common diseases in dairy farming, can cause a reduction in milk production and quality, negatively impacting the economic benefits of livestock farms [2,3]. Therefore, the timely detection of dairy mastitis is essential for dairy farming to prevent the further deterioration of udder health. Subclinical mastitis does not exhibit visible signs of inflammation but may cause an increase in somatic cell count (SCC) in milk. Clinical mastitis is a more severe form of mastitis that exhibits visible signs of inflammation and may also cause changes in the milk and discomfort in the cow.

To achieve this goal, several detection methods and pieces of equipment, such as SCC, the California mastitis test (CMT), and the milk pH test, have been studied. However, these methods require the measurement of physicochemical components in cow milk samples using specialized instruments under strict environmental conditions, and the overall operation process is tedious and costly [4,5,6].

Infrared thermograph technology (IRT) is a non-invasive and non-destructive method of body temperature measurement that has been used by numerous researchers as a physiological and pathological diagnostic tool [7,8,9]. Zaninelli et al. [10] confirmed the significant positive correlation between the udder surface temperature of cows and SCC by analyzing thermal infrared images and set the threshold for subclinical mastitis classification at 200,000 cells/mL SCC. George et al. [11,12] approved that ocular surface temperature (OST) measured by IRT can be used as a temperature indicator of cows. In addition, Sathiyabarathi et al. [13] used IRT technology to observe the body temperature and udder skin surface temperature (USST) of dairy cows. The results showed that the temperature of the diseased part was 0.8 °C higher than the body temperature. Consequently, IRT can be used as the detection indicator for mastitis in dairy cows.

In this context, Metzner et al. [14] used different geometric tools to manually measure key parts of the thermal infrared image of dairy cows and ROC analysis to find the best temperature measurement method. To improve the accuracy of mastitis detection in cows, Zhang Xudong et al. [15] proposed a cow’s eye and udder position detection algorithm based on threshold segmentation and support vector machines to achieve the automatic detection of cow mastitis. However, the average detection accuracy of mastitis was only 60.4%. In summary, the limited automation and poor detection performance of these methods make them unsuitable for the large-scale practical needs of detecting mastitis in dairy cows.

In recent years, with the rapid development of deep learning in computer vision, convolutional neural networks (CNNs) have achieved significant success in target detection scenarios with complex backgrounds [16,17,18,19]. Wang Yanchao et al. [20] proposed an improved YOLO v3-tiny target detection algorithm to further enhance the accuracy and efficiency of eye and udder positioning. The accuracy of mastitis detection was 77.3%. In another study, to promote the detection accuracy of key parts of dairy cows, Xudong et al. [21] proposed an EFMYOLOv3 deep learning algorithm based on thermal infrared images, which achieved an average positioning accuracy of 96.8% and an average classification accuracy of 83.33% for mastitis. To reduce the influence of external factors, Wang et al. [22] combined the temperature difference between the left and right udder skin surfaces, as well as the temperature difference between the OST and the USST. The detection accuracy rate of cow mastitis disease was 85.71%. However, some areas near key parts of dairy cows, such as the high-temperature areas of the cow’s abdomen, cleavage, and inner thigh, may lead to inaccurate target detection, resulting in errors in temperature extraction and affecting the accuracy of dairy cow mastitis detection.

**Figure 1:**
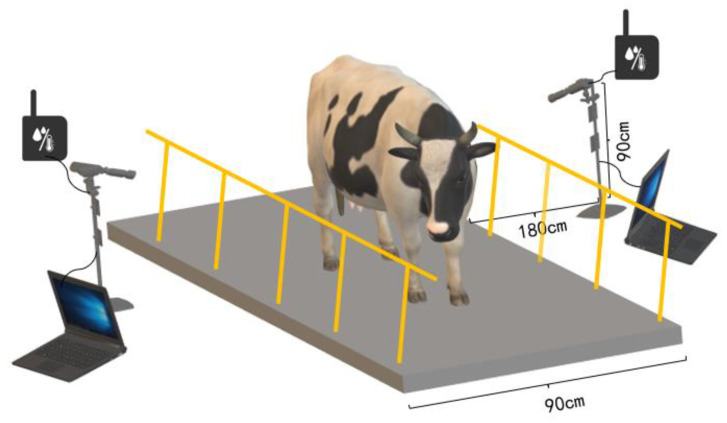
Schematic diagram of the cow thermal infrared acquisition system.

We proposed a CLE-UNet model to achieve the accurate automatic segmentation of cow eyes and udders to solve the above problems and further promote the detection accuracy of cow mastitis. The overall process is shown in Figure 1. We selected UNet [23] as the algorithm framework to segment key parts of cows and introduced an efficient channel attention (ECA) mechanism in the down-sampling layer of UNet’s encoder section, which allows the network to focus on more useful channel features during training [24]. A new centroid loss function was proposed to guide and constrain model optimization during training, bringing the predicted value closer to the position of the real value by calculating the Euclidean distance between the predicted centroid position and the centroid position of the real mask. Finally, we proposed an ellipse fitting operation step that improves the segmentation effect of cow eyes by ellipticizing the cow eyes in the segmentation results. In the conducted experiments, the CLE-UNet model was employed to extract temperature data from key parts of dairy cows for mastitis detection. The findings reveal the viability and efficacy of the proposed method within the scope of automated cow mastitis detection.

## 2. Materials and Methods

### 2.1. Dataset

#### 2.1.1. Experimental Device

**Figure 2:**
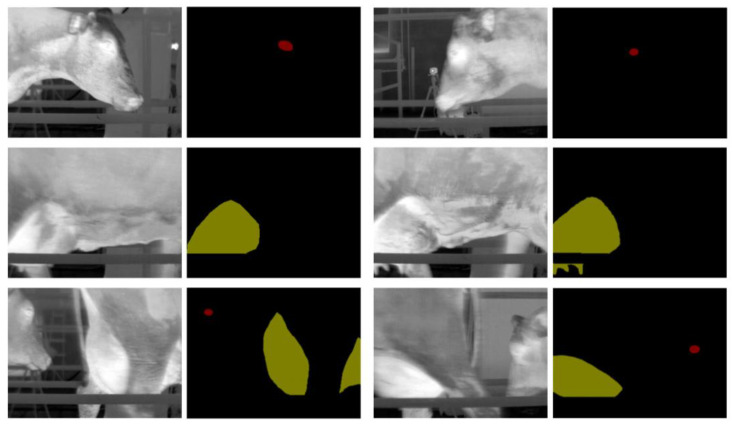
Rendering of data preprocessing.

Experimental data were collected in the indoor milking hall of Dadi Herd Dairy Cattle Farm in Yanqing District, Beijing, China, at 8–10 a.m. in July 2021. As illustrated in Figure 2, cows entered the milking hall through a 90 cm wide channel, and a fixed data acquisition device—FLIR A310 thermal imaging camera (FLIR Systems, Wilsonville, OR, USA)—was symmetrically installed on both sides of the channel. We used the HOBO U14-001 (FLIR Systems, Wilsonville, OR, USA) intelligent temperature and humidity recorder to calibrate the temperature readings obtained from the FLIR Tools software by measuring the ambient temperature and relative humidity around the thermal imager. To ensure that the thermal imaging camera can capture the major parts of cows, the linear distance between the base of the camera bracket and the edge of the channel was 180 cm, and the vertical height of the lens from the ground was set at 90 cm. The emissivity of the thermal imager was set at 0.98, in agreement with previous relevant studies [25]. A total of 180 thermal infrared videos (video format is SEQ) of cows were collected, and their milk samples were tested for SCC. It should be noted that if the SCC value is greater than or equal to 200,000 cells/mL, that side of the cow’s udder was considered to have high SCC, consistent with the related research [13]. We tested milk samples for SCC from each quarter of each cow’s udder and also matched the location of each cow’s udder in the thermal images with their SCC results manually to ensure accuracy. It is important to note that our data collection process was designed to avoid any disruption to the cows’ daily activities, ensuring that their welfare was not compromised.

#### 2.1.2. Dataset Descriptions

**Figure 3:**
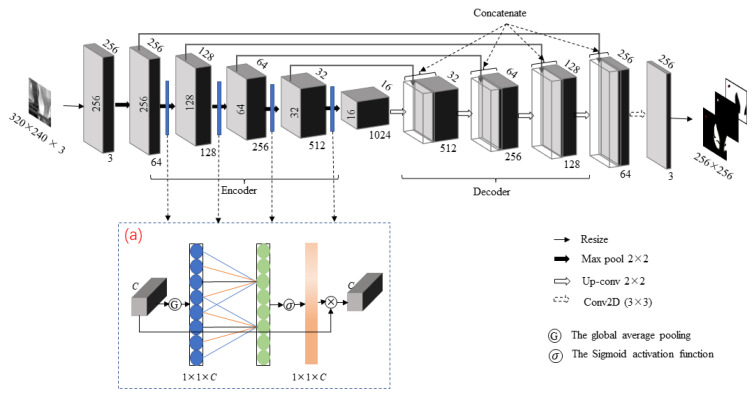
The architecture of CLE-UNet. (**a**) ECA attention mechanism module.

The experimental object of this article was a group of 180 lactating Holstein cows. Each cow was considered as two different acquisition objects due to the different conditions of its left and right udder. Therefore, thermal infrared videos of a total of 360 cows were obtained. These thermal infrared videos were subjected to the following processing steps. Firstly, the thermal infrared videos of 360 cows were divided into JPG images at a rate of 15 frames per second, with a resolution of 320 pixels × 240 pixels. The original images were manually filtered to obtain 2400 thermal infrared images of dairy cows after removing images with severe occlusion, pixel blur, and incorrect posture. Additionally, the Labelme software was used to manually segment the eyes and the udders of the cows and to label them as different categories, resulting in a JSON format file containing the label location and category. Finally, the JSON file was converted into a PNG-formatted mask image for training the deep learning model. The datasets were then divided into training, verification, and test sets in the ratio of 3:1:1. Partial images and corresponding mask images after the above preprocessing are shown in Figure 3. Consequently, the thermal infrared images of cows contain three types of labels, and their representative meanings are shown in Table 1.

### 2.2. Experimental Setup

Table 2 presents the hardware platform configuration used for experimental training and testing. The model was trained for 100 epochs with a learning rate of 0.0001 and the Adam optimizer. Due to GPU memory limitations, a batch size of 8 was used during training. Each input image had a resolution of 256 × 256. The training parameters for the other models were the same.

### 2.3. CLE-UNet Network Model

Aiming at the problems of weak contours and weak contrast in thermal infrared images of dairy cows, this study proposes the CLE-UNet model based on UNet, which is more suitable for practical tasks of segmenting key parts in thermal infrared images of dairy cows. The model structure is illustrated in Figure 3, showing the size of the feature map and the number of channels after the input data pass through each layer. The main improvements include adding an ECA attention mechanism to each layer of the encoder to automatically detect regional features in important channels. Additionally, during network training, a new centroid loss function was used to calculate the Euclidean distance between the centroids of the segmented region and the target region. Ultimately, the accuracy of eye segmentation was improved by ellipse fitting processing operations.

#### 2.3.1. Backbone

Considering the speed and effect, we choose UNet as the main backbone to extract the features of the image. The architecture of the UNet model consists of two parts: the encoder and the decoder [23]. The encoder part consists of convolution layers (with a convolution core size of 3 × 3) and down-sampling layers (a global maximum pooling layer with a step size of 2) for feature extraction. The decoder combines the underlying location information with the underlying semantic information using up-sampling to restore the original resolution of the feature map and jump connections.

#### 2.3.2. Attention Mechanism

ECA (efficient channel attention) is a lightweight channel attention mechanism that uses one-dimensional convolution to capture local cross-channel interactions, capturing inter-channel dependencies without dimensionality reduction operations, resulting in a significant improvement in model performance [24]. The structure of the ECA attention mechanism module is shown in Part (a) in Figure 3. The specific process of this module is as follows: to start with, a global average pooling operation is performed on the input feature map, changing its size to 1 × 1 × *C*, where *C* is the number of channels for the input feature. Then, the weight value of each channel is calculated by convolution operation and sigmoid activation function. The one-dimensional convolution kernel size was set to 5. Finally, the weight is multiplied by the original feature map to obtain the final output feature map. From the whole process, it can be seen that the computational process of the ECA attention mechanism is relatively simple, with low module complexity and little impact on the speed and performance of the network. Therefore, in the encoder part of the UNet model, an ECA attention mechanism is added before each down-sampling layer to improve the performance and generalization of the network by dynamically adjusting the weight of channels, removing redundant information and enhancing useful information and thus improving the segmentation accuracy of the model.

#### 2.3.3. A New Centroid Loss Function

The Lovasz-Softmax loss is a loss function designed for intersection over union (IoU) optimization, introduced in CVPR 2018, which ultimately achieved the best results on both Pascal VOC and Cityscapes datasets [26]. Experiments have demonstrated that the Lovasz-Softmax loss outperforms other loss functions in multi-category segmentation tasks.

**Figure 4:**
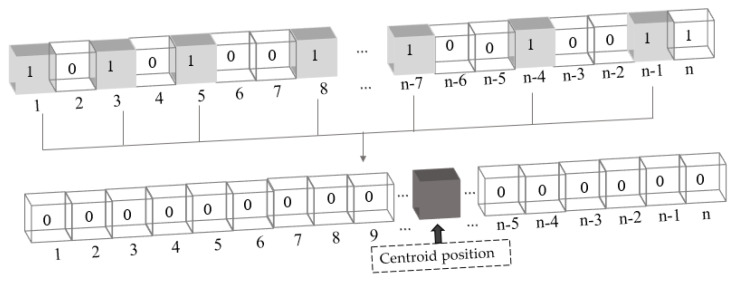
Schematic diagram of centroid position calculation.

In this study, due to the natural walking process of cows, the location of key parts was unstable and susceptible to significant changes. Consequently, we proposed a new centroid loss function to align the training results of the model more closely with the actual label location. This approach involves adding the Softmax activation function to the last layer of the model. Furthermore, the feature size of each channel was reshaped into a matrix with a width of 1 and a length equal to the input feature map size. We then employed the same processing method to calculate the centroid positions of the predicted results and the actual label, with the centroid position being a real number between 0 and n, and its calculation formula is shown in Equation (1), where C is the centroid position. Finally, the Euclidean distance between the centroid positions of the predicted result and the real label was computed and mapped to a range of 0 to 1 using the sigmoid function, ensuring that the distance metric is on the same scale as the Lovasz-Softmax loss value. By minimizing the centroid distance, the model can produce better segmentation results. Figure 4 presents a schematic diagram for calculating the centroid position of the feature map, where n is the length of the feature map. The centroid loss calculation formula is shown in Equation (2), and the final loss function expression used in this experiment is presented in Equation (3).

(1) $$C=\frac{\sum_{i=1}^{n}{(x}_{i}\times w_{i})}{\sum_{i=1}^{n}w_{i}}$$

(2) $$L_{z}=\left|C_{P}-C_{T}\right|$$

(3) $$L_{new}=\sigma \left(\frac{1}{1+e^{-L_{z}}}\right)+L_{Lovasz-Softmax}$$

where n is the size of the feature map, $w_{i}$ represents the weight of the *i*th position in the feature map, $x_{i}$ is the subscript of the *i*th position in the feature map, $C_{P}$ is the centroid positions of the predicted result, $C_{T}$ is the centroid positions of the real label, $L_{Lovasz-Softmax}$ denotes the loss value of Lovasz-Softmax, σ is the Sigmoid loss function, $L_{z}$ is the centroid loss value, and $L_{new}$ is the new centroid loss value.

#### 2.3.4. Ellipticization Processing

**Figure 5:**
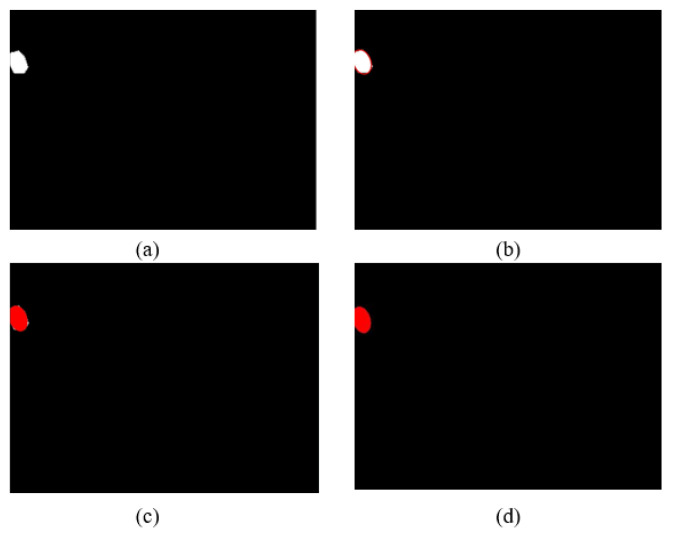
Effect of ellipse fitting processing. (**a**) Segmentation results of cow eyes; (**b**) Elliptic boundary fitting; (**c**) Ellipse interior filling result; (**d**) Segmentation results of cow eyes after ellipticization processing.

Owing to the blurred boundary of the key parts in the thermal infrared image of dairy cows, the prediction results of the model are prone to errors. According to observation and common sense, it was found that the eye region of a cow closely resembles an elliptical shape. Therefore, this study proposed an additional processing step after image segmentation—an ellipse-fitting operation—to improve the segmentation effect of the cow’s eye region. The specific steps are as follows: firstly, we need to calculate the center position of the segmented image in the cow’s eye region, the length of the long axis, the length of the short axis, and the rotation angle of the ellipse; then, according to the above indicators, we fitted the ellipse to the cow’s eye, filled the inner region of the ellipse, and remove the remaining parts outside the boundary. Finally, we took it as the final segmentation result. Figure 5 demonstrates the effect of the ellipse-fitting process. From the figure, it can be observed that the ellipse fitting of the cow’s eye region preserves the position and angle of the original region and effectively removes burrs from the edges.

For the known set of irregular graph edge points in the model output, we used the least squares method to fit a quadratic curve to obtain relevant parameters of the ellipse, such as the central position of the ellipse, the length of the semi-major axis a, the length of the semi-minor axis, and the tilted angle. The standard elliptic equation formula can be converted to a rotated elliptic equation formula by coordinate rotation transformation. The elliptic equation formula, coordinate rotation transformation and rotated elliptic equation are shown in Equations (4)–(6).

(4) $$\frac{x^{2}}{a^{2}}+\frac{y^{2}}{b^{2}}=1$$

(5) $$\left[\begin{array}{cc} \cos\beta & -\sin\beta \\ \sin\beta & \cos\beta \end{array}\right]\left[\begin{array}{c} x\\ y \end{array}\right]=\left[\begin{array}{c} x\cos\beta -y\sin\beta \\ x\sin\beta +y\cos\beta \end{array}\right]$$

(6) $$\frac{{\left(x\cos\beta -y\sin\beta \right)}^{2}}{a^{2}}+\frac{{\left(x\sin\beta +y\cos\beta \right)}^{2}}{b^{2}}=1$$

where *a* is the semi-major axis of the ellipse; *b* is the semi-minor axis of an ellipse; *x* represents the abscissa of a point on the ellipse; *y* is the ordinate of a point on the ellipse; and β is the clockwise tilt of the ellipse.

### 2.4. Model Evaluation Indicators

#### 2.4.1. Image Segmentation Effect Evaluation Indicators

In this experiment, we used IoU as a segmentation performance evaluation index for the CLE-UNet model. By calculating the ratio of intersection and union between the real and predicted values, the matching degree between the segmented image and the real label was evaluated. The IoU calculation formulas for each category and all categories are shown in Equations (7) and (8).

(7) $$IoU=\frac{SP\cap ST}{SP\cup ST}$$

(8) $$MIoU=\frac{1}{K+1}\left(\sum_{i=1}^{K+1}\frac{{SP}_{i}{\cap ST}_{i}}{{SP}_{i}{\cup ST}_{i}}\right)$$

The thermal infrared image includes a background, with a total of K+1 categories, where K is the number of target categories, ${SP}_{i}$ is the prediction result for the ith category, and ${ST}_{i}$ is the actual label of the ⅈth category.

#### 2.4.2. Temperature Acquisition and Diagnostic Criteria for Cow Mastitis

According to relevant research, when a cow is suffering from subclinical mastitis, the temperature in the udder area is 0.8 °C higher than the normal body temperature, and the temperature of the cow’s eyes can represent its core body temperature [13,27]. Therefore, in this experiment, the conditions for determining the mastitis of the cow can be expressed as Equation (9). The maximum temperature data of the eyes and udders were extracted from all thermal infrared images of each cow, and based on this, the eye and the udder temperature description parameters were established as Equations (10) and (11).

(9) $$S_{mastitis}=\left\{\begin{array}{cc} 0 & T_{udder}-T_{eye}\leq 0.8\\ 1 & T_{udder}-T_{eye}>0.8 \end{array}\right.$$

(10) $$T_{eye}=\underset{n\in \left[1,N_{e}\right]}{Max}\left(\underset{i,j\in \left[1,S_{e}\right]}{{Max}_{n}}\left(T\left(x_{i},y_{j}\right)\right)\right)$$

(11) $$T_{udder}=\underset{m\in \left[1,N_{u}\right]}{Max}\left(\underset{i,j\in \left[1,S_{u}\right]}{{Max}_{m}}\left(T\left(x_{i},y_{j}\right)\right)\right)$$

where $T_{eye}$ is the eye temperature of the cow, which is its core body temperature; $T_{udder}$ is the udder temperature of the cow; and $S_{mastitis}$ is the diagnostic result of cow mastitis, where a value of 0 indicates “normal” and a value of 1 indicates “illness”. $N_{e}$ is the number of thermal infrared images containing the eye area of the cow, $N_{u}$ is the number of thermal infrared images of the cow containing the udder area, $S_{e}$ is the number of elements in the cow’s eye temperature matrix in the thermal infrared image, and $S_{u}$ is the number of elements in the cow’s breast temperature matrix in the thermal infrared image.

#### 2.4.3. Evaluation Indicators of Dairy Cow Mastitis

To verify the effectiveness of the algorithm, it is necessary to use performance evaluation indicators to test the model. In this study, we use the Sensitivity, Recall, Specificity, and F1 score as evaluation indices. Their calculation formulas are shown in Equations (12)–(15). Sensitivity is a measure of the proportion of *TP* that are correctly identified by the model. Recall is a measure of the proportion of actual positive cases that are correctly identified by the model. Specificity is a measure of the proportion of *TN* that are correctly identified by the model. The F1 score is a measure of the overall performance of the model. Accuracy is a measure of how well the model correctly identifies both *TP* and *TN*.

(12) $$Sensitivity=\frac{TP}{TP+FP}$$

(13) $$Recall=\frac{TP}{TP+FN}$$

(14) $$Specificity=\frac{TN}{TN+FP}$$

(15) $$F1=2\times \frac{Sensitivity\times Recall}{Sensitivity+Recall}$$

where TP is the true positives, TN is the true negatives, FP is the false positives, and FN is the false negatives.

## 3. Results and Discussion

### 3.1. The Segmentation of Key Parts of Dairy Cows Based on CLE-UNet Model

**Figure 6:**
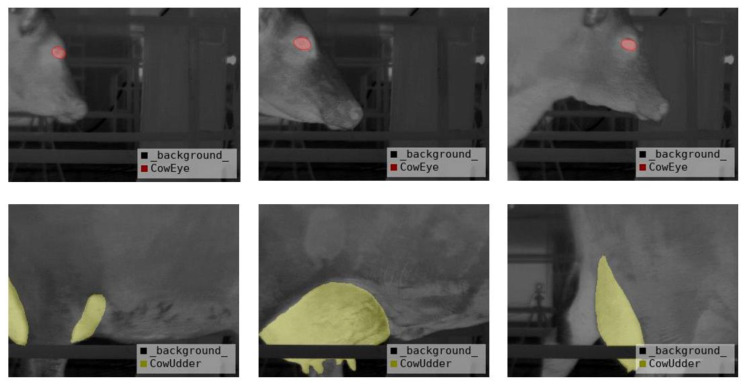
Segmentation effects yielded for key parts of dairy cows.

Table 3 presents the results of comparing the performance of different improved methods based on the UNet deep learning model. As shown in the table, the IoU of background category segmentation in thermal infrared images of cows was higher than 96.63%, and the improvement space was limited. On the ground of the network characteristics of UNet multiscale fusion, the ECA channel attention mechanism is introduced in the feature extraction. Compared with the original UNet model, the MIoU of the UNet-1 model is improved by approximately 1.14%. To further improve the segmentation accuracy, the UNet-2 model utilized a new centroid loss function, resulting in an MIoU improvement of about 1.2%, and the eye’s IoU was improved by 2.35% compared to the UNet-1 model. Based on the similarity between the shape of the cow’s eye and the ellipse, we proposed that the CLE-UNet was based on the UNet-2 model. By employing the eye ellipse-fitting processing operation, the cow eye’s IoU of the CLE-Une model increased by about 8.67% compared to the UNet-2 model. Consequently, the MIoU of the CLE-UNet image segmentation algorithm proposed in this study reached 89.32%, with the image background, cow eyes, and cow udders having an IoU of 97.7%, 86.59%, and 83.66%, respectively, and a segmentation rate of 0.049 s per image. Compared to the original UNet model, the cow’s eye IoU increased by 12.7%, the udder’s IoU increased by 4.5%, and the total MIoU increased by 6.09%. Overall, experiments have shown that the CLE-UNet model has a highly accurate segmentation performance. Figure 6 displays the segmentation results of the CLE-UNet model, where red represents the eye region of the cow, green represents the udder region of the cow, and the rest represents the background.

### 3.2. Performance Comparison among Different Segmentation Models

To verify the segmentation performance of the CLE-UNet model for key parts of dairy cows in thermal infrared images, we compared it with five classical deep learning segmentation models: DeepLab v3+ [28], SegNet [29], PSPNet [30], UNet [23], and UNet++ [31]. The experimental results are presented in Table 4. From the table, it can be observed that among these models, DeepLab v3+ exhibited excellent segmentation performance, with its MIoU being approximately 1.75 percentage points higher than that of the SegNet model. However, due to the complexity of the DeepLab v3+ model, its segmentation speed of 0.087 s per frame was relatively the slowest. While the segmentation effect of the PSPNet model was similar to that of the SegNet model, its speed was about 0.019 s slower than that of the SegNet. The UNet model achieved a good balance between segmentation effectiveness and speed. Therefore, the UNet model was chosen as the base model for the experiment. The test set of the CLE-UNet model exhibited an MIoU of 89.32 and a frame rate of 0.049 s per frame. Compared with the UNet model, the CLE-UNet model has improved the eye and udder segmentation effects by 12.7% and 4.5%, respectively, with a slightly reduced speed. This proves that the CLE-UNet model proposed in this study has excellent segmentation performance, and the ellipse-fitting operation proposed for eye segmentation and the novel loss function proposed for segmented target location have strong feasibility.

**Figure 7:**
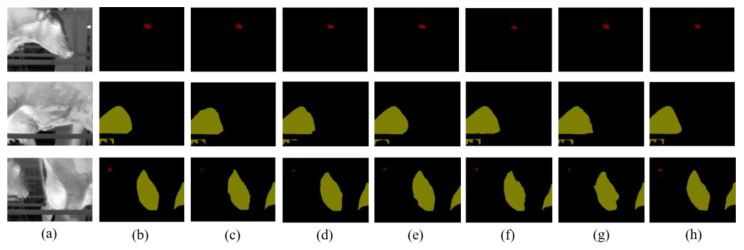
The segmentation effect of key parts of dairy cows in different models. (**a**) Original image. (**b**) Actual label. (**c**) DeepLab v3+ segmentation effect. (**d**) SegNet segmentation effect. (**e**) PSPNet segmentation effect. (**f**) UNet segmentation effect. (**g**) UNet++ segmentation effect. (**h**) CLE-UNet segmentation effect.

Figure 7 depicts the segmentation effect of the above-mentioned models on the thermal infrared images of dairy cows. It is evident from the figure that in the presented models, the CLE-UNet model exhibits a smooth segmentation region, with the segmentation position being in close proximity to the tag position. Moreover, the eye segmentation image obtained after ellipse-fitting processing is more similar to the actual tag image.

### 3.3. Evaluation of the Dairy Cow Mastitis Recognition Result

#### 3.3.1. Result of the Dairy Cow Mastitis Recognition of CLE-UNet Model

**Figure 8:**
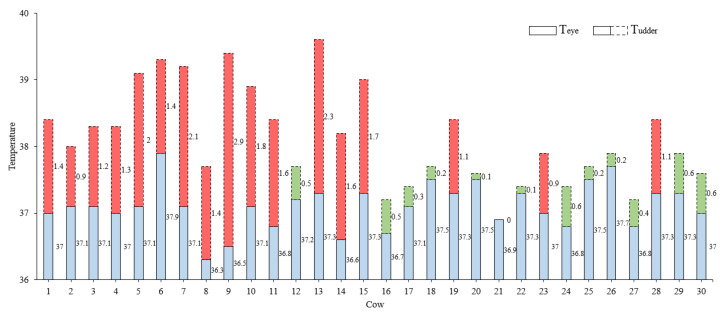
The ocular surface temperature and udder skin surface temperature of dairy cows in the test set. The red: the USST of subclinical mastitis affected is higher than 0.8 °C compared to OST. The green: the difference between normal USST and OST is less than 0.8 °C. The blue: the cow’s OST.

To verify the performance of the CLE-UNet semantic segmentation cow mastitis detection algorithm, we randomly selected 30 cows (15 normal and 15 sick) from the original dataset for testing and strictly distinguished their left and right sides. In addition, the SCC results were used as the verification set. In the test set, we extracted the temperature of key parts of the cow based on the segmentation results obtained using the CLE-UNet algorithm. The temperature data are presented in Figure 8, where the height of the solid rectangular bar indicates the ocular surface temperature of the cow, and the height of the dotted rectangular bar represents the udder skin surface temperature of the cow. If the temperature difference between the two exceeds 0.8 °C, the rectangular bar is marked in red, and if it is lower than 0.8 °C, the bar is marked in green. The results of cow mastitis detection are presented in Table 5. According to formulas 11 to 14, the accuracy rate, sensitivity rate, recall rate, specificity, and F1 value of the algorithm for the detection of cow mastitis were calculated to be 86.67%, 82.35, 93.33, 80%, and 87.5%, respectively. The experimental results indicate that utilizing the CLE-UNet for the thermal infrared image segmentation of cows can enhance the accuracy of key part positioning, extract more realistic temperatures, and effectively improve mastitis detection in cows.

#### 3.3.2. Comparison of the Effectiveness of Related Detection Methods for Dairy Cow Mastitis

Table 6 compares the effectiveness of relevant cow mastitis detection methods based on thermal infrared images. It is evident from the table that the CLE-UNet image segmentation algorithm, constructed in this paper, exhibits a superior classification accuracy for cow mastitis and uses smaller pixels of experimental data compared to other relevant research. This proves that the CLE-UNet cow mastitis detection algorithm, based on deep learning image segmentation technology proposed in this study, can perform high-precision automatic segmentation of key parts of cows, reduce temperature extraction errors, and effectively improve the detection accuracy of cow mastitis.

## 4. Conclusions

In this study, we proposed a novel CLE-UNet semantic segmentation algorithm for mastitis detection in dairy cows, which achieved high accuracy and rapid segmentation speeds. Our results demonstrate the potential of the CLE-UNet algorithm for improving mastitis detection in dairy cows and highlight the need for complementary diagnostic methods and advanced data collection equipment to further enhance the accuracy and specificity of mastitis diagnosis.

While our study has shown promise in using thermal infrared data for mastitis detection, we recognize that further improvements are necessary to fully realize the potential of this method. Factors such as skin color, manure coverage, and hairiness can impact the accuracy and reliability of thermographic results and, therefore, must be addressed to improve the method’s effectiveness. To address these limitations, future research could consider using additional cameras or a camera under the platform to better analyze the inferior udder total surface and manually segmenting the udder parts on a teat basis to better analyze the thinner part of the udder wall. Additionally, automating these improvements through temperature recording could help reduce the impact of these factors on the accuracy and reliability of thermographic results.

Nonetheless, our study represents an important step towards more effective and efficient mastitis detection in dairy cows, which could ultimately lead to improved animal health and milk production. Further research in this area is warranted to fully understand the potential of the proposed method and to explore its practical applications for dairy farm managers and veterinarians.

## Figures and Tables

**Table 1:** Label description.

Category No.	Label	Description
0	background	Background
1	cowEye	Cow eyes
2	cowUdder	Cow udder

**Table 2:** The hardware platform configuration table.

Configuration	Parameter
CPU	Intel (R) Core (TM) i9-10900K CPU
GPU	NVIDIA GTX3060
Development environment	Python 3.8.11
Operating system	Ubuntu 18.04
Operating framework	PyTorch 1.9.0
Accelerate environment	CUDA 11.4 + CUDNN 8.11

**Table 3:** Comparison among the results of each improved method.

Method	①	②	③	Background_IoU (%)	Eye_IoU (%)	Udder_IoU (%)	MIoU (%)
UNet	-	-	-	96.63	73.89	79.16	83.23
UNet-1	√	-	-	96.81	75.57	80.72	84.37
UNet-2	√	√	-	97.45	77.92	81.35	85.57
CLE-UNet	√	√	√	97.70	86.59	83.66	89.32

① ECA attention mechanism. ② Centroid loss function. ③ Ellipticization of the eye.

**Table 4:** Performance comparison of different segmentation models.

Algorithm	Background_IoU (%)	Eye_IoU (%)	Udder_IoU (%)	MioU (%)	Time per Frame (s)
DeepLab v3+	97.46	74.44	81.26	84.39	0.087
SegNet	95.02	73.67	79.24	82.64	0.025
PSPNet	95.99	71.88	80.07	82.65	0.044
UNet	96.63	73.89	79.16	83.23	0.032
UNet++	96.82	74.38	78.06	83.09	0.052
CLE-UNet	97.70	86.59	83.66	89.32	0.049

**Table 5:** Results of mastitis detection of dairy cow.

Cow	RFID	Left or Right	Algorithm Detected Results	Somatic Cell Count Results
1	19,235	Right	Mastitis (1)	Mastitis (1)
2	18,165	Left	Mastitis (1)	Mastitis (1)
3	19,173	Left	Mastitis (1)	Mastitis (1)
4	8110	Right	Mastitis (1)	Mastitis (1)
5	19,039	Left	Mastitis (1)	Mastitis (1)
6	18,088	Left	Mastitis (1)	Mastitis (1)
7	18,026	Left	Mastitis (1)	Mastitis (1)
8	15,214	Right	Mastitis (1)	Mastitis (1)
9	19,016	Left	Mastitis (1)	Mastitis (1)
10	8138	Right	Mastitis (1)	Mastitis (1)
11	8048	Right	Mastitis (1)	Mastitis (1)
12	8123	Right	Normal (0)	Mastitis (1)
13	17,106	Left	Mastitis (1)	Mastitis (1)
14	7205	Right	Mastitis (1)	Mastitis (1)
15	19,152	Right	Mastitis (1)	Mastitis (1)
16	19,124	Right	Normal (0)	Normal (0)
17	18,183	Left	Normal (0)	Normal (0)
18	19,121	Left	Normal (0)	Normal (0)
19	8055	Right	Mastitis (1)	Normal (0)
20	7129	Right	Normal (0)	Normal (0)
21	19191	Right	Normal (0)	Normal (0)
22	19,141	Left	Normal (0)	Normal (0)
23	7212	Left	Mastitis (1)	Normal (0)
24	1073	Right	Normal (0)	Normal (0)
25	18,293	Right	Normal (0)	Normal (0)
26	19,082	Left	Normal (0)	Normal (0)
27	17,106	Right	Normal (0)	Normal (0)
28	7199	Left	Mastitis (1)	Normal (0)
29	15,018	Left	Normal (0)	Normal (0)
30	19,070	Right	Normal (0)	Normal (0)

**Table 6:** Comparison of the effectiveness of related detection methods for dairy cow mastitis.

Detection Method	Image Resolution	Sensitivity (%)	Specificity (%)	F1 (%)	Accuracy (%)
YOLO V3-tiny [20]	640 × 480	69.23	66.67	78.26	77.30
EFMYOLOv3 [21]	640 × 480	75.00	76.47	82.76	83.33
CLE-UNet	320 × 240	82.35	80.00	87.50	86.67

## Data Availability

The data presented in this study are available on request from the corresponding author. The data are not publicly available due to being part of an ongoing study.
